# How big is the myelinating orchestra? Cellular diversity within the oligodendrocyte lineage: facts and hypotheses

**DOI:** 10.3389/fncel.2014.00201

**Published:** 2014-07-28

**Authors:** Giulio Srubek Tomassy, Valentina Fossati

**Affiliations:** ^1^Department of Stem Cell and Regenerative Biology, Harvard UniversityCambridge, MA, USA; ^2^The New York Stem Cell FoundationNew York, NY, USA

**Keywords:** oligodendrocytes, cellular diversity, myelin sheath, CNS development, oligodendrocyte progenitors, cellular identity

## Abstract

Since monumental studies from scientists like His, Ramón y Cajal, Lorente de Nó and many others have put down roots for modern neuroscience, the scientific community has spent a considerable amount of time, and money, investigating any possible aspect of the evolution, development and function of neurons. Today, the complexity and diversity of myriads of neuronal populations, and their progenitors, is still focus of extensive studies in hundreds of laboratories around the world. However, our prevalent neuron-centric perspective has dampened the efforts in understanding glial cells, even though their active participation in the brain physiology and pathophysiology has been increasingly recognized over the years. Among all glial cells of the central nervous system (CNS), oligodendrocytes (OLs) are a particularly specialized type of cells that provide fundamental support to neuronal activity by producing the myelin sheath. Despite their functional relevance, the developmental mechanisms regulating the generation of OLs are still poorly understood. In particular, it is still not known whether these cells share the same degree of heterogeneity of their neuronal companions and whether multiple subtypes exist within the lineage. Here, we will review and discuss current knowledge about OL development and function in the brain and spinal cord. We will try to address some specific questions: do multiple OL subtypes exist in the CNS? What is the evidence for their existence and those against them? What are the functional features that define an oligodendrocyte? We will end our journey by reviewing recent advances in human pluripotent stem cell differentiation towards OLs. This exciting field is still at its earliest days, but it is quickly evolving with improved protocols to generate functional OLs from different spatial origins. As stem cells constitute now an unprecedented source of human OLs, we believe that they will become an increasingly valuable tool for deciphering the complexity of human OL identity.

## Introduction

One of the main challenges of modern neurobiology is discovering how cellular diversity in the brain emerges during development. Together with synaptic plasticity, neuronal diversity is the *mantra* that supports a functional complexity that would otherwise be difficult to explain. In the last decade we can find many examples that have taught us about the importance of dissecting the nervous system into small groups of neuronal subtypes, and, sometimes, even subtypes of subtypes. Years of studies on the generation and specification of neuronal identity in the spinal cord, retina and cerebral cortex have revealed common and divergent paths that lead to the establishment of extremely intricate networks (Livesey and Cepko, 2001; Arlotta et al., 2005; Migliore and Shepherd, 2005; Tomassy et al., 2010; Belgard et al., 2011). Understanding how millions of different neurons develop, integrate and eventually function as a whole is not just a mere intellectual exercise aimed at satisfying our *Faustian* aspirations, but it may also have direct consequences for our clinical approach to disease. A very recent example of this comes from studies on Rett syndrome, a neurodevelopmental disorder caused by mutations in the gene Mecp2 (Bienvenu and Chelly, 2006; Chahrour and Zoghbi, 2007). Although Mecp2 is expressed by many different types of neuronal and non neuronal cells (Kishi and Macklis, 2004; Caballero and Hendrich, 2005), its loss apparently does not affect all brain areas in a similar way (Tudor et al., 2002; Chahrour and Zoghbi, 2007).

However, neurons are not the only citizen in the nervous system where, although the proportion is still controversial, a significant fraction is represented by glial cells (Pakkenberg and Gundersen, 1988; Azevedo et al., 2009; Kandel et al., 2012). In the central nervous system (CNS), glial cells come in three flavors: astrocytes, oligodendrocytes (OLs) and microglia (Verkhratsky and Butt, 2007; Stern, 2010). The many roles for this “adhesive” type of cells have recently begun to attract a well-deserved attention (the number of glia-centered articles tripled in the last 30 years according to NCBI). Despite the fact that neurons are critically supported by glial cells in the generation of active synapses and without glia would not communicate in a timely manner, just to name some of the glial contribution to neuronal function (Baumann and Pham-Dinh, 2001; Clarke and Barres, 2013), neuroscience, as its name suggests, is still strongly neuron-centric. However, neuronal diversity would mean nothing without the 24/7 support of their glial partners. In light of all this, one obvious question stands up for an answer: are glial cells as diverse as neurons? In other words how many types of astrocytes exist? How many OLs? And if multiple types exist, how much diversity exists among the progenitors that give rise to glial cells? Astrocytes seem to constitute a fairly heterogeneous population of cells (Zhang and Barres, 2010), but our knowledge about OL still lags far behind. Here we will focus on this type of glial cells, and their progenitors oligodendrocyte progenitor cells (OPCs), that are in charge of making the myelin sheath. Currently, there is much debate ongoing on whether OPCs constitute a homogeneous population of cells or whether different subtypes exist, as would be suggested based on different molecular and functional parameters (Gensert and Goldman, 2001; Dimou et al., 2008; Rivers et al., 2008; Lin et al., 2009; Psachoulia et al., 2009). Here, we will first review the different developmental origins of OPCs and the multiple lineages to which they belong. We will then review what is known about OL ability to produce the myelin sheath. We will then consider the evidences about some of the “other” functions of OL and how they may suggest the existence of multiple cell types, or subtypes. As stated before, these are not trivial issues, whose implications may also have clinical relevance, considering how many developmental and degenerative diseases affect the maturation and survival of OLs and their fatty membrane (Di Rocco et al., 2004; Trapp and Nave, 2008). We will end our article by reviewing current technical advances in generating OPCs and OLs in a dish, for therapeutic purposes, and we will speculate on the implications of a yet unexplored heterogeneity on such studies and on our understanding of human myelin pathologies.

## Space oddity. Different sites of origin for the myelinating cells

Although the spatial origin of OLs has long been debated (Richardson et al., 2006), a consensus has now been reached about the dual nature of these cells as deriving from both ventral and dorsal domains of the neuraxis. Here, we will briefly review our current knowledge about four regions of the murine CNS, namely the neocortex, the olfactory bulbs (OB), the cerebellum and the spinal cord, and we will try to summarize and highlight common themes and divergent paths.

## Neocortex

**Figure 1:**
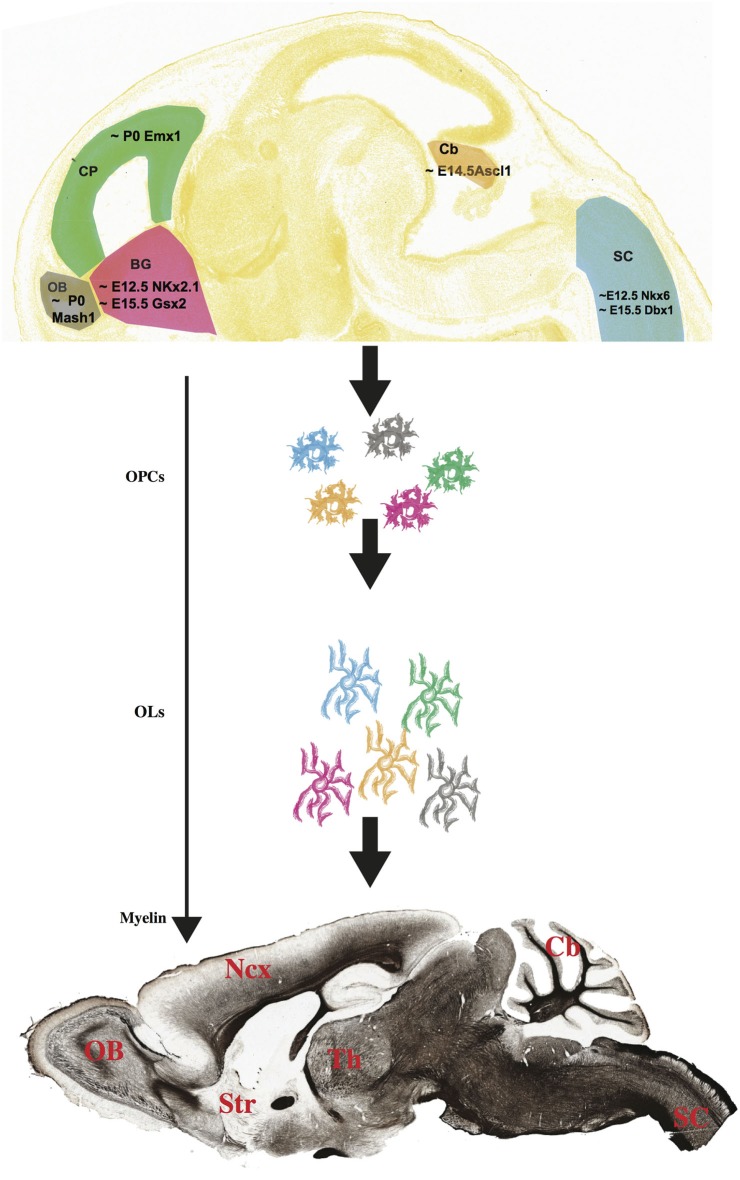
**Multiple progenitors, one myelin?** (Top) OPCs have different birthdates and spatial origins in the embryonic and postnatal mouse brain and spinal cord (Image credit: Allen Institute for Brain Science, www.alleninstitute.org). How diverse are these multi-lineage OPC cells, and do they give rise to different groups of myelinating OLs? The myelin stained sagittal section on the bottom has been downloaded from the Brain Architecture Project (http://mouse.brainarchitecture.org) and licensed under a Creative Commons (CC) Attribution-ShareAlike 3.0 Unported License (http://creativecommons.org/licenses/by-sa/3.0/). OB, olfactory bulb; CP, cortical plate; BG, basal ganglia; Cb, cerebellum; SC, spinal cord; Ncx, neocortex; Str, striatum; Th, thalamus. Scale bar, 1 mm.

The migratory fluxes that contribute to the formation of the neocortical wall have been matter of extensive research over the last decades. During development, two major streams contribute to the building of the cortical structure: a radial one, perpendicular to the pia, that starts in the proximity of the ependymal surface and a tangential one, that runs over long distances starting in the germinal zones of the ventral telencephalon. Classically, cortical projection neurons, born in the VZ and subventricular zone (SVZ) of the cortical primordium, migrate radially, while GABAergic cortical interneurons migrate tangentially from the medial and caudal ganglionic eminences (MGE, CGE) of the ventral telencephalon and enter the cortical primordium through two main tangential streams (Marín and Rubenstein, 2001, 2003). Although it was thought for a long time that cortical OLs originated only in the ventral telencephalon (Tekki-Kessaris et al., 2001), Kessaris et al. (2005) subsequently demonstrated the existence of three different migratory paths, each belonging to a different cell lineage (Figure 1). Two of these migratory paths, corresponding to Gsx2^+^ and Nkx2.1^+^ cell lineages, originate in the lateral ganglionic eminence (LGE) and in the MGE, respectively, while a third one, Emx1^+^ lineage, originate within the cortical anlage itself. In the adult cortex, the Gsx2 and the Emx1 waves are the only two lineages contributing to myelination, as the Nkx2.1-derived OPCs apparently do not give rise to any myelinating OL, and they disappear after the first week of postnatal life (Kessaris et al., 2005).

## Olfactory bulbs

Very little is known about the origin of OLs of the OB. Experimental evidences including heterotopic, heterocronic transplantations as well as genetic fate mapping point at an intrinsic source of these cells, although additional and more conclusive analysis has not been conducted yet (Spassky et al., 2001). In the postnatal mouse brain, NG2^+^ progenitor cells in the SVZ migrate through the rostral migratory stream (RMS) and give rise to OB oligodendrocytes as well as neurons (Aguirre and Gallo, 2004). In addition, it has been shown that these SVZ-derived OPCs belong to the Mash1^+^ lineage (Figure 1). Indeed, Mash1 mutant mice showed a dramatic decrease of OPCs in the OBs (Parras et al., 2004).

## Cerebellum

In the chicken brain, cerebellar OLs originate in the parabasal bands of the ventral mesencephalum, from which they migrate tangentially and enter the developing cerebellum through the velum medullare (Mecklenburg et al., 2011). In the murine cerebellum, Ascl1-expressing OLs originate outside the cerebellar germinal epithelium, although the exact region still needs to be identified (Buffo and Rossi, 2013; Figure 1). As in the neocortex and in the spinal cord (see below), a dorsal, intracerebellar source of OLs cannot be ruled out and some evidence suggest that at least some OLs may indeed originate within the local germinal zones: by injecting a beta-galactosidase expressing retrovirus into post-natal deep germinal zone of the rat cerebellum, Zhang and Goldman showed that many labeled cells gave rise to OLs. However, this endogenous production of cerebellar OLs ceases by the third week of post-natal life (Zhang and Goldman, 1996; Grimaldi et al., 2009; Sudarov et al., 2011; Zhang et al., 2011).

## Spinal cord

In the spinal cord two regions have been recognized as sources of OLs. The first one, lying within the expression territory of the transcription factors Nkx6.1 and Nkx6.2 (Cai et al., 2005; Fogarty et al., 2005; Vallstedt et al., 2005) is located in the pMN domain of the embryonic cord, where the transcription factor Olig2 regulates the generation of both OLs and motor neurons (Rowitch et al., 2002) and accounts for 85% of all spinal cord OLs (Richardson et al., 2006). However, OLs are still generated in the Nkx6 knockout spinal cord, suggesting that other sources exist in the developing cord (Cai et al., 2005; Vallstedt et al., 2005). Indeed, fate mapping experiments using a Dbx1-driven Cre reporter line confirmed the existence of a smaller but consistent group of cells that are born in the dP3 to dP5 dorsal domains of the cord (Fogarty et al., 2005; Figure 1).

## Time is on my side. Multiple developmental stages of oligodendrogenesis

The emerging picture shows that OLs originate in many different regions along the rostro-caudal and dorso-ventral axes of both brain and spinal cord. Furthermore, these cells are generated at different times during both embryonic and post-natal development. In the spinal cord, generation of ventral progenitors starts around embryonic (E) day 12.5, while the first dorsal progenitors are generated at approximately E14.5 (Cai et al., 2005). By then, the only OPCs that can be found in the neocortex belong to the Nkx2.1 lineage, while Gsx2^+^ OPCs have not reached the cortical plate yet (Kessaris et al., 2005), and Olig2^+^ cells can be already detected in the developing cerebellum (Buffo and Rossi, 2013). Meanwhile, Emx1^+^ progenitors in the developing neocortical wall are still producing projection neurons of the upper layers, with their gliogenic potential still on standby (Angevine and Sidman, 1961; Gorski et al., 2002) and waiting to give rise to OLs only at birth (around P0) (Kessaris et al., 2005; Figure 1). Aside from the Emx1^+^ lineage, that produces neocortical OLs postnatally, all other OPCs are born embryonically, and their “myelination potential” (the actual formation of a compact myelin envelope) is “released” gradually, following a spatio-temporal sequence that is a developmental signature for any given species (Baumann and Pham-Dinh, 2001). In mice, this sequence starts in the spinal cord at birth and follows a caudorostral direction toward the brain, with the neocortex being the last region to be myelinated. In humans, myelination begins at midgestation in the spinal cord, and continues for at least the first two decades of life (Baumann and Pham-Dinh, 2001).

## The song remains the same. Many oligodendrocytes, one myelin?

Does this spatial and temporal heterogeneity produce a heterogeneous population of OLs? If we look at neurogenesis, the birthdate and place of origin of a neuron are typically linked to its identity and function. A classical example comes from the neocortex. Here, glutamatergic projection neurons and GABAergic interneurons are born in the germinal zones of two distant regions of the developing telencephalon, the dorsal neocortical epithelium and the MGE and CGE of the ventral telencephalon, respectively (Marín and Rubenstein, 2003; Xu et al., 2004; Butt et al., 2005; Molyneaux et al., 2007). MGE-derived interneurons comprise two types of cells with very distinct molecular and functional features, i.e., Nkx2.1^+^/Parvalbumin^+^ and Nkx2.1^+^/Somatostatin^+^ interneurons (Butt et al., 2008), that in turn can be further subdivided into different subtypes, mainly based on their electrophysiological properties (Ma et al., 2006; Runyan et al., 2010). Since a cohort of neocortical OPCs also belongs to the Nkx2.1^+^ lineage (Corbin et al., 2001; Marín and Rubenstein, 2001; Kessaris et al., 2005), it is tempting to hypothesize that these cells might also share some level of heterogeneity, like their neuronal counterparts in the lineage. Remaining within the borders of this audacious analogy, Emx1^+^ glutamatergic neuron identity is strongly correlated to their birthdate, such that neurons born first, between E12.5 and E13.5 become corticofugal projection neurons of the deep layers of the neocortex, while late born cells become commissural projection neurons of the superficial layers (Molyneaux et al., 2007). Thus, an obvious question is whether Emx1^+^ neocortical OLs born at different times also have different cellular identities and/or functions. Also, how different are the Emx1^+^ OLs from the Gsx2^+^ OLs? Nicoletta Kessaris and William Richardson crossed a Sox10-lox-GFP-poly(A)-lox-DTA mouse line with either a Gsx2-Cre or an Emx1-Cre effector line. The conditional excision of the GFP activated the DTA in selected cells and killed them (Kessaris et al., 2005). With this elegant approach, the authors showed that ablation of one type of OLs, e.g., Gsx2-derived or Emx1-derived, does not affect the final number of Sox10^+^ cells (i.e., all OLs) nor the level of myelination in every region analyzed, including the neocortex. Thus, when one precursor pool of OLs is lost, the other one may compensate for its absence, implying that these cells are fully interchangeable and functionally equivalent. On the other hand, however, the same group has recently demonstrated that, in the spinal cord, dorsally and ventrally-derived OLs are not equally able to myelinate the dorsal corticospinal tract (CST), which runs in the dorsal funiculus of the cord. The authors showed that the CST is mainly myelinated by dorsally-derived OLs, that within the first 2 months of post-natal life outnumber and almost completely replace their ventrally-derived partners (Tripathi et al., 2011). Thus, in the spinal cord, OLs with different spatial and temporal origins may be differentially able to myelinate neighboring axons, suggesting that at least part of their identity must be affected by their developmental history. Although the reasons for this have not been investigated, one possibility is that dorsal and ventral OLs may express different “codes” of molecules (e.g., membrane receptors) that may govern interactions with specific neuronal subtypes (e.g., corticospinal motor neurons). Indeed, it is widely accepted that OL development and myelin biogenesis are strongly influenced by neuron-derived signals (Barres and Raff, 1999; Stevens et al., 2002; Nave and Salzer, 2006; Taveggia et al., 2010; Wake et al., 2011). However, one may speculate that for the same reasons, different OLs myelinating sequential segments of one single CST axon, must share some common traits, regardless of their origin or birthdate. This is puzzling, considering the remarkable length of the CST and the different regions of the brain and spinal cord that it runs through (Arlotta et al., 2005; Martin, 2005). Further research on the interactions between multiple OLs lineages and long axons like the CST are certainly desirable, and will further expand our understanding of neuron-oligodendrocyte biology.

**Figure 2:**
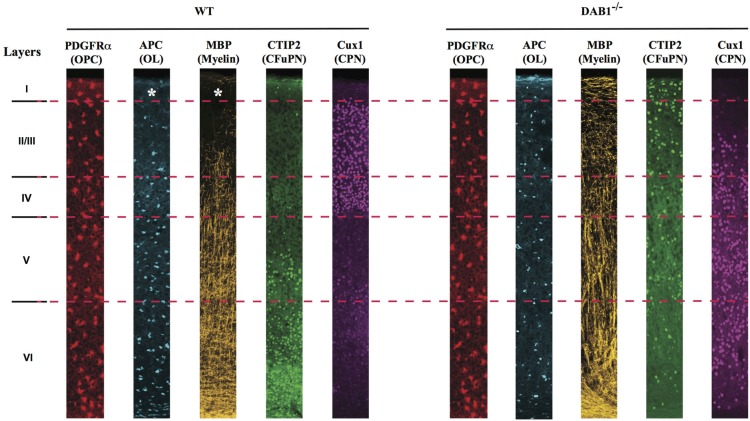
**Neocortical OPCs of different layers are not equally able to give rise to myelinating OLs.** Schematic representation of laminar distribution of PDGFRα^+^ OPCs, APC^+^ OLs, MBP^+^ myelin, CTIP2^+^ corticofugal (CFuPN) and Cux1^+^ commisural (CPN) projection neurons in the neocortex of (left) WT and (right) Dab1^−/–^ mice. OPCs are uniformly distributed in all layers of the cortex, but only in layer IV to VI they generate mature, myelin-forming OLs. In the nearly inverted cortex of the DAB1^−/–^ mice, both mature OLs and myelin are found in the most superficial part of the cortex, suggesting that repositioning of deep layer CFuPN has shaped the myelin profile of the neocortex.

Within the neocortical gray matter, the scenario is even more compelling, as suggested by a very recent paper from the laboratory of Paola Arlotta, in which we have shown that while Pdgfrα^+^ OPCs populate all layers of the neocortex, their ability to generate mature APC^+^/Plp1^+^ OLs is dependent on their laminar position within the neocortex; accordingly, the amount of myelin found in the superficial layers is dramatically lower as compared to the deep layers (Tomassy et al., 2014; Figure 2). What are the reasons for this uneven distribution of myelinating cells in the neocortical wall? Superficial layers (II-IV) mostly contain commissural pyramidal neurons (CPN), that connect the two hemispheres of the brain as well as different cortical areas within the same hemisphere. Deep layer V and VI, instead, contain corticofugal pyramidal neurons (CFuPN), connecting the cortex with subcerebral and subcortical targets (Molyneaux et al., 2007, 2009). We showed that the layer-specific ability of neocortical OPCs to give rise to myelinating OLs is affected by the neuronal subtype present in their immediate proximity. Specifically, by changing the position of deep layer pyramidal neurons within the cortical wall, OLs redistribute and the myelination profile of the cortex changes accordingly; for example, in the Dab1^−/–^ neocortex, where layers are nearly inverted, (i.e., deep layer neurons are located in the upper part of the cortex, while upper layer neurons are located in the deep layers) (Sweet et al., 1996; Ware et al., 1997), both OLs and myelin lose their gradient profile and instead cover the full extent of the cortex (Figure 2; Tomassy et al., 2014). Thus, our study suggests that different combinations of neuron-oligodendrocyte interactions may exist in different layers of the cortex; however, also layer-specific cell-autonomous differences among neocortical OPCs and/or OLs may not be ruled out. As a matter of fact, regional differences in OPCs behavior have been previously reported by several groups. Marsupials are a great model to study oligodendrogenesis, because of the extended development of their CNS, and a temporal analysis of CNPase expression on glia revealed a heterogeneous distribution of CNPase^+^ cells over time, with only a transient expression in certain areas such as the optic pathway (Barradas et al., 1998). Magdalena Götz and Leda Dimou used genetic fate mapping in mice to follow the fate of Olig2^+^ cells in the adult brain and showed that these cells generate myelinating OLs in the white matter, but remain as NG2^+^ postmitotic cells in the gray matter (Dimou et al., 2008). They later went on and performed homo and heterotopic transplantation of traceable cells from adult gray and white matter to demonstrate that there are intrinsic differences between the progenitors residing in these two different environments; more specifically, only white matter-derived cells can efficiently generate myelinating OLs in both white and gray matter, while cells from the gray matter have a lower differentiation potential and fail to differentiate in a non-supportive environment such as the gray matter (Viganò et al., 2013). A plausible way to explain those regional differences is to assume that the OL population is heterogeneous. The existence of different types of OLs has actually been suggested from the very first work of Del Rio Ortega, which described four types of OLs, identified by morphology and number of processes (del Rio Hortega, 1928). Later studies highlighted a correlation between the different types of OLs and the size of the axons myelinated by them, showing that small/highly ramified type I-II OLs, expressing carbonic anhydrase II (CAII), preferentially myelinate small caliber axons, while type III-IV larger OLs, with only one or two processes and negative for CAII myelinate large fibers (Butt et al., 1995). In addition, OLs have been divided into subsets according to the distribution of myelin basic proteins within their cytoplasm, nucleus and plasmalemma (Hardy et al., 1996). More recently, the development of sophisticated imaging techniques has been helpful to dissect OL heterogeneity; for example, using a computerized cell tracing system, Murtie et al. (2007) identified in the mouse frontal cortex an unknown subpopulation of OLs with numerous processes and short myelin internodes.

All together, these studies strongly support the existence of at least some level of heterogeneity within the oligodendrocyte lineage.

Understanding such diversity is crucial, giving the direct clinical implications that this might have: for example, it is well known that in multiple sclerosis (MS) there are two types of lesions, depending on the stage of disease, those with depletion of OPCs and those with normal number of OPCs that fail to differentiate and myelinate (Chang et al., 2002; Franklin, 2002). Although multiple reasons may account for these differences, one possibility is that different types of OPCs behave differently upon demyelinating conditions; this has been also suggested by studies showing that, upon demyelination of the rat spinal cord, two different populations exist, dividing and non-dividing OPCs, that respond differently to the demyelinating event (Keirstead et al., 1998). Also, the analysis of periventricular white matter injury (PWMI) in the ovine model brought to light the existence of areas more vulnerable to ischemia, due to a lower degree of maturity of the residing glia cells, suggesting that a more extensive maturation of OLs could protect infants from PWMI (Riddle et al., 2006). To note, an interesting study emphasizes the concept of regional differences in relation to cell replacement therapies, since different areas of the brain show specific sensitivity to irradiation, resulting in different levels of OPCs depletion and consequently in a variable degree of repopulation of the irradiated areas (Irvine and Blakemore, 2007).

## Hey hey, what can I do? What do oligodendrocytes (and their progenitors) do, apart from making myelin?

Apart from their neurotransmitter system of choice and the networks where they integrate, all neurons have the same basic constituents: a cell body, an axon and multiple dendrites, and more importantly an excitable membrane, which is probably the most peculiar feature of this cell type. If we look at OLs, the most peculiar feature of these cells is their ability to wrap their plasma membrane around axons and form the myelin sheath. However, what else do we know about them? Are there properties by which one could distinguish cell subtypes or functional specializations?

OPCs have been described to share some “neuron-like” electrical features, such as expression of ion channels and ability to fire single action potentials, *in vitro* and *in vivo* (Kettenmann et al., 1984; Barres et al., 1990a,b; Bergles et al., 2010; Almeida and Lyons, 2013; Sun and Dietrich, 2013). In the white and gray matter of the mouse forebrain, OPCs have distinct physiological properties and express different profiles of Na^+^ and K^+^ channels. More importantly, a group of cells in the gray, but not white matter, responds with single, TTX-sensitive spikes upon depolarizing current injections. This spiking population of cortical NG2^+^ cells also expresses functional AMPA receptors (Chittajallu et al., 2004). This is similar to what has been found in the rat hippocampus, where OPCs receive both glutamatergic as well as GABAergic synaptic inputs (Bergles et al., 2000; Lin and Bergles, 2004). These data seem to suggest that these physiological properties are not a common trait of the OL lineage, but rather a specialization of a subtype of cells of the gray matter, that may distinguish them from OPCs located in the white matter. However, in the white matter of the early postnatal rat cerebellum, two distinct populations of morphologically identical OPCs were found: again, one population expressed Na^+^ and K^+^ channels, received both inhibitory as well as excitatory synaptic inputs and, more importantly, was able to fire action potentials upon stimulation. The other population instead was not able to generate action potentials and did not receive any synaptic input (Káradóttir et al., 2008). Thus, the distinction between spiking and non-spiking cells may not be relevant in distinguishing white versus gray matter OPCs, but rather a specific feature that can be utilized to identify two functionally different subtypes, regardless of their location within the CNS. Interestingly, although physiologically active, spiking OPCs maintain their mitotic status, suggesting that neuronal inputs may be required to control or modulate their proliferative activity (Ge et al., 2009). Indeed, it has been demonstrated that neuronal activity influences oligodendrogenesis and myelination both *in vitro* and *in vivo* (Demerens et al., 1996; Wake et al., 2011; Gibson et al., 2014). All together, these data suggest that two subtypes of OPCs may exist, both in the gray as well as in the white matter, that can be distinguished based on their membrane properties and ability to spike action potentials. Interestingly, one of the symptoms of human oligodendrogliomas, a primary tumor of the brain (Harvey and Cushing, 1926; Russell and Rubinstein, 1959; Canoll and Goldman, 2008), is the frequent occurrence of epileptic seizures, likely due to the ability of these tumor cells to generate action potentials (Patt et al., 1996). Although one possibility is that these cells infiltrate the tissue and produce seizures by changing the microenvironment around neighboring neurons, another intriguing possibility is that this type of tumor exclusively may originate from the spiking, but not from the non-spiking subtype of OPCs.

## Tomorrow never knows. Understanding human OLs through pluripotent stem cells differentiation

The ultimate goal of basic research, apart from pure scientific curiosity, is to translate what we learn into practical tools that we can use to treat and possibly cure human diseases. Since one of the current main challenges of myelin research is to being able to produce functional OPCs and OLs *in vitro*, for therapeutic use (Goldman et al., 2012), understanding what are the common features that univocally identify these cells, as well as those that may distinguish distinct subtypes, is compelling and worth a concerted effort of the whole scientific community.

**Figure 3:**
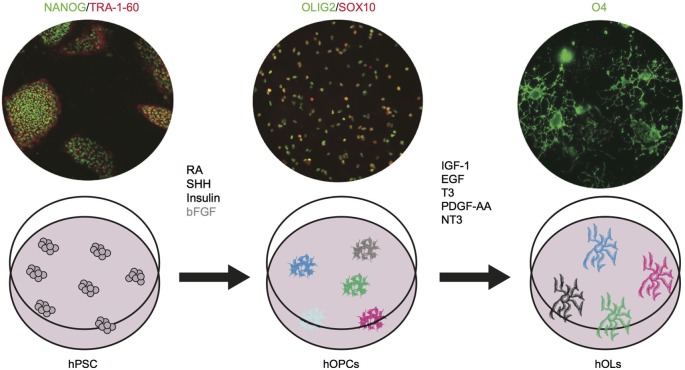
**Human OPCs and OLs can be differentiated** ***in vitro*** **from pluripotent stem cells.** hPSC are shown as NANOG^+^ (green)/TRA-1-60^+^(red); hOPCs as OLIG2^+^(green)/SOX10^+^(red); and hOL as O4^+^ cells (green). Images are based on results in press in Stem Cell Reports (Douvaras et al., 2014). The critical agents that promote oligodendrocyte differentiation have been identified. We showed that bFGF (shown in gray) is dispensable. Future studies will investigate whether the cells generated in the dish constitute one homogenous population or different subtypes. RA: retinoic acid, SHH: sonic hedgehog, bFGF: basic fibroblast growth factor, IGF-1: insulin like growth factor-1, EGF: epidermal growth factor, T3: triiodothyronine, PDGF-AA: platelet derived growth factor, NT3: neurotrophin 3.

During the last two decades, the stem cell field has developed at a remarkable pace. Through the discovery of human embryonic stem cells (ESC) first (Thomson et al., 1998), and the generation of human induced pluripotent stem (iPS) cells via genetic reprogramming of somatic cells, less than 10 years later (Takahashi et al., 2007), we now own an unprecedented tool for studying human embryonic development and for generating all types of cells of the body. Pioneering studies on ESC differentiation clearly showed that embryonic development can be successfully recapitulated in a step-wise manner *in vitro* and that the fundamental pathways of lineage commitment are largely conserved from mouse to human (D’Amour et al., 2005; Kennedy et al., 2007). Those principles have been applied to oligodendrocyte development and resulted in several differentiation protocols, in which human pluripotent stem cells (hPSC, encompassing both ESC and iPSC) could be efficiently committed to an oligodendrocyte fate by patterning with critical molecules identified through studies on rodents (Nistor et al., 2005; Izrael et al., 2007; Hu et al., 2009; Figure 3).

A combination of retinoic acid (RA), insulin, insulin-like growth factor (IGF-1), triiodothhyroidin (T3) hormone, epidermal growth factor (EGF) and basic fibroblast growth (bFGF) was required to achieve the first oligodendrocyte differentiation from hESCs (Nistor et al., 2005). Platelet derived growth factor (PDGF-AA) and neurotrophin3 (NT3) were also added later on to drive maturation of OPCs to OLs (Hu et al., 2009). RA has been extensively used *in vitro* to mimic caudalization of neural tissues (Wichterle et al., 2002). Insulin and IGF-1 act as survival factors for oligodendrocyte progenitors and mature OLs (Barres et al., 1992). T3 plays a critical role in various stages of oligodendrocyte development, by promoting generation and expansion of early progenitors, differentiation to mature oligodendrocyte, and myelination (Rodríguez-Peña, 1999). EGF and FGF promote OPC generation and proliferation (McKinnon et al., 1990; Gonzalez-Perez and Alvarez-Buylla, 2011). Further attempts at obtaining an efficient *in vitro* differentiation protocol focused on spinal cord development. The progression toward mature OLs was followed through the sequential upregulation of OLIG2, NKX2.2, SOX10, PDGFRα, O4, and MBP (Izrael et al., 2007). Induction through RA and sonic hedgehog (SHH) signaling recapitulated *in vitro* the patterning of neuroepithelial cells to OLIG2^+^ progenitors of the pMN domain (Hu et al., 2009). Interestingly, while this strategy confirmed that the transcriptional network regulating oligodendrocyte development is largely conserved among mammals, the study uncovered differences between human and mouse. First, NKX2.2 is expressed in human cells *in vitro* immediately after OLIG2 and before PDGFRα, as it occurs in the chick (Xu et al., 2000) and in the mouse hindbrain development (Vallstedt et al., 2005), but not in the mouse spinal cord (Qi et al., 2001). Second, bFGF in human cultures plays two distinct roles at different stages of the differentiation process, increasing the number of OLIG2^+^ progenitors by preventing motor neuron differentiation and subsequently inhibiting SHH signaling and the differentiation from OLIG2^+^/NKX2.2^+^ pre-OPCs to SOX10^+^ OPCs. Finally, a long transition time (around 9 weeks) from pre-OPCs to OPCs appears to be a distinctive feature of human cultures, while it is not seen during mouse ESCs differentiation (Najm et al., 2011). This could reflect the slower temporal progression in human fetal development compared to mouse (Jakovcevski et al., 2009), but it could also be—at least in part—due to suboptimal culture conditions, as shown by our more recent protocol, in which the transition phase from pre-OPCs to OPCs is significantly shortened. Interestingly, we have also found that exogenous FGF signaling was dispensable in our cultures (Douvaras et al., 2014). With the discovery of iPS cells, *in vitro* differentiation studies largely moved to the optimization of the available protocols to extend the reproducibility to hiPSC lines (Pouya et al., 2011; Wang et al., 2013; Douvaras et al., 2014). Studies with iPS cells have confirmed that patterning with RA and SHH are an effective strategy to recapitulate oligodendrogenesis of the spinal cord, but to date the characterization of OLs differentiation has been purely restricted to well established markers such as SOX10, PDGFRα, NG2, O4, O1, MBP. One may wonder whether, in reality, we are generating a mixed population of OLs, and whether multiple subtypes of OPCs and OLs exist and can be recognized *in vitro*. Following the example of neuronal studies, single cell gene expression profiling could help addressing this question (Citri et al., 2012).

A recent study attempted for the first time to generate and characterize OPCs and OLs from both spinal cord and forebrain. Once more, the lesson was learned from mouse embryonic development, where FGF signaling, via *Fgfr1* and *Fgfr2* was identified as critical to generate *Pdgfrα*^+^, *Olig2*^+^ OPCs from ventral forebrain. The translation of those findings to human differentiation *in vitro* allowed the successful generation of human forebrain GSX2^+^/NXX2.1^+^ OPCs through induction with bFGF and SHH (Stacpoole et al., 2013). The next step will be to investigate whether forebrain OLs exhibit functional features that are distinct from their spinal cord counterparts. Interestingly, the same study provided the first evidence that hOPCs, similarly to mouse and rat OPCs, can be divided into spiking and nonspiking, and future studies will possibly identify molecular and functional differences associated with these subtypes. While our understanding of oligodendrogenesis improves by means of embryological studies in animal models, we will soon develop increasingly optimized protocols for differentiating hPSC towards OPCs from different developmental origins and with different identities. As a result, these *in vitro* systems will provide a novel compelling platform to thoroughly dissect human OL diversity. We envision that forthcoming studies of human OLs in a dish will lead the search for the answer. As stem cells constitute an unprecedented source of human OLs, they will become a fundamental tool for deciphering the complexity of oligodendroglia identity. The more we will learn from basic developmental biology and physiological studies, the more we will be able to “make” cells *in vitro* that will closely resemble their *in vivo* counterpart.

## Conclusions

How much diversity exists within the oligodendrocyte lineage? Once more, the question is more practical than it may sound, with profound, direct implications into our clinical approach. From a “myelination perspective”, OPCs from one region of the CNS can substitute for populations derived from other regions (e.g., neonatal forebrain SVZ could generate OLs when injected into the neonatal cerebellum), suggesting that these cells have a “default” myelinogenic potential that doesn’t change with the environment (Milosevic et al., 2008). One-sixty years after the term “nervenkitt” was coined (Virchow, 1859), we have just started to turn our magnifying glasses toward the right direction and recognize these cells as something more than just cerebral glue. Today’s technology will certainly help us accelerating this process and we can envision that the immediate future will bring us new knowledge as well as new concepts and ideas. Modern high-throughput genome and transcriptional profiling techniques (Cahoy et al., 2008; Wang et al., 2009; Shapiro et al., 2013) combined with the latest imaging (Lichtman and Denk, 2011) and electrophysiology tools (Fenno et al., 2011) will certainly boost our understanding of the diversity of OPCs and OLs in the CNS. Thus, it doesn’t matter whether OLs perform as a symphony orchestra, a solo or a rock band, relax and enjoy the music: their show has just started.

## Conflict of interest statement

The authors declare that the research was conducted in the absence of any commercial or financial relationships that could be construed as a potential conflict of interest.
